# Protein Biomarkers in Chronic Kidney Disease in Children—What Do We Know So Far?

**DOI:** 10.3390/jcm12123934

**Published:** 2023-06-08

**Authors:** Agnieszka Rybi Szumińska, Anna Wasilewska, Monika Kamianowska

**Affiliations:** Department of Peadiatrics and Nephrology, Medical University of Bialystok, Waszyngtona 17, 15-297 Bialystok, Poland; anna.wasilewska@udsk.pl (A.W.);

**Keywords:** chronic kidney disease, children, protein biomarker, end-stage kidney disease

## Abstract

Chronic kidney disease (CKD) in children is a major concern of medical care and public health as it is related to high morbidity and mortality due to progression to end-stage kidney disease (ESKD). It is essential to identify patients with a risk of developing CKD to implement therapeutic interventions. Unfortunately, conventional markers of CKD, such as serum creatinine, glomerular filtration rate (GFR) and proteinuria, have many limitations in serving as an early and specific diagnostic tool for this condition. Despite the above, they are still the most frequently utilized as we do not have better. Studies from the last decade identified multiple CKD blood and urine protein biomarkers but mostly assessed the adult population. This article outlines some recent achievements and new perspectives in finding a set of protein biomarkers that might improve our ability to prognose CKD progression in children, monitor the response to treatment, or even become a potential therapeutic target.

## 1. Introduction

CKD in children is a progressive condition leading to kidney failure with other dangerous consequences, including high cardiovascular risk, mineral and bone disorder, developmental delay, and growth retardation [1,2]. In the literature, reliable data on the epidemiology of CKD in children are deficient and come mostly from kidney replacement therapy (KRT) registers (ESPN/ERA Registry in Europe or USRDS in the USA) [3,4]. Only a few studies have assessed CKD stages 2–5 in children with the use of estimated glomerular filtration rate (eGFR) according to KDIGO guidelines, and almost none investigates chronicity criterion (3 months of observation) to exclude transient fluctuations in kidney function. Nevertheless, data from the last 3 decades estimate the prevalence of pediatric CKD between 30 and 50 per million age-related population (pmarp) per year [5].

It should be emphasized that the etiology of pediatric CKD differs significantly from the adult population, where it is mainly secondary to hypertension or diabetes. The pattern of causes of progressive kidney impairment in children in developed countries responsible for about two-thirds of all cases is congenital anomalies of the kidneys and the urinary tract (CAKUT), including obstructive uropathy, reflux nephropathy or hypo- and dysplastic kidneys, and glomerulopathies (steroid-resistant nephrotic syndrome and other chronic glomerulonephritides). Similarly, the reduction in the number of nephrons associated with low-birth weight and small-for-gestational-age infants, as well as the rising incidence of pediatric obesity, are very important predisposing factors of the development of CKD [6,7,8].

According to the definition by KDOQI in 2002 and endorsed in 2012 by KDIGO, CKD in children is characterized by persistent kidney damage with normal eGFR (stage 1) or a drop in eGFR below 60 mL/min/1.73m^2^ lasting for at least 3 months [9,10]. Diagnosis of CKD in the pediatric population is still based on the assessment of conventional biomarkers: serum creatinine with eGFR and proteinuria. They are far from the ideal kidney function indicators in children while increasing relatively late in the course of the kidney impairment [11]. It has to be emphasized that despite ongoing injury, the kidney can maintain GFR to some extent by hyperfiltration and compensatory hypertrophy of the glomeruli [12,13]. Each of these traditional indicators has other important limitations. Serum creatinine concentration is affected by the patient’s race, gender, muscle mass, hydration or drugs, and therefore it only partially reflects kidney function [14]. Proteinuria is present in a persistent tubular injury, glomerular hyperfiltration or primary glomerular disorders, inflammation and tubulointerstitial fibrosis [15]. There are several pediatric studies that confirm the association between the intensity of proteinuria expressed as a urinary protein/creatinine ratio and the progression of CKD [16,17,18]; however, none of them indicates proteinuria as an early biomarker of CKD.

The process of CKD begins with an initiating factor causing the chronic injury of affected cells [19,20]. Regardless of the primary cause, the progression of CKD is quite similar and leads to tubulointerstitial disease. Subsequently, nephrons undergo stress resulting in the activation of inflammatory response and maladaptive tissue repair. With time, there is a progressive nephrons loss, developing interstitial fibrosis and compensatory hyperfiltration of healthy glomeruli [16,20]. It increases the permeability of the glomerular membrane, resulting in proteinuria and activation of the renin–angiotensin system with an increase in systemic blood pressure. Those two adverse consequences additionally escalate existing chronic kidney injury [17]. This pathophysiologic model of CKD is accompanied by the presence of different protein markers in plasma or urine that may act as regulators or be products of particular stages of the process.

There is a great need to establish a set of newer kidney biomarkers useful in the early detection and prognosis of the progression of CKD in children. We have to emphasize that CKD in children is not a homogeneous disorder, and proposed biomarkers may also reflect the condition responsible for the CKD.

A biomarker should identify a normal or pathogenic process or a response to therapeutic intervention. Ideally, its measurement should be non-invasive, easy to perform, sensitive and specific, repeatable and low-cost [21,22]. The Food and Drug Administration—National Institute of Health Biomarker Working Group describes types of biomarkers using terms diagnostic, prognostic, predictive, useful for monitoring the disease or for a pharmacodynamic response, safe and assessing risk or disease susceptibility [23]. Protein biomarkers are mainly mediators or byproducts of the processes involved in the pathway of CKD. Some of them are known, but many are still waiting to be detected and tested. We still do not have a newer protein biomarker validated for commercial use to diagnose or assess the progression of CKD in children. There are few candidates for this role. 

This paper aims to gather evidence from the literature, especially from the last 5 years, regarding the most common protein biomarkers of CKD in children and present them as a review.

## 2. Materials and Methods

### 2.1. Search Protocol

The analysis was performed with the guidelines of the updated PRISMA 2020 statement (Preferred Reporting Items for Systematic Reviews and Meta-Analyses) [24]. The review was based on three databases: PubMed, Web of Science and Embase. All records between January 2018 and January 2023 were included with the search strategy using MeSH (Medical Subject Heading) terms and keywords for the description of population and intervention with the help of the Boolean operators ”or”, “and”. We used the combination of: “chronic kidney disease” AND “children” AND “protein markers” AND “blood” OR “urine”.

The inclusion criteria were as follows: (1) human research; (2) study group <18 years old and >1 month old; (3) clinical diagnosis of CKD or condition leading to CKD; (4) “new” protein markers obtained in blood or urine (creatinine, proteinuria or albuminuria did not meet the criterion of a “new” marker; (5) cohort and case-control investigations; (6) articles published in English in a peer-reviewed journal. The exclusion criteria were as follows: (1) animal research; (2) newborns or adults included; (3) no specific diagnosis of CKD; (4) presence of other severe diseases or treatment that might interfere with the results of obtained protein kidney markers; (5) no control group to compare samples or control group was not precisely chosen; (6) studies not in English; (7) full text not available.

**Figure 1:**
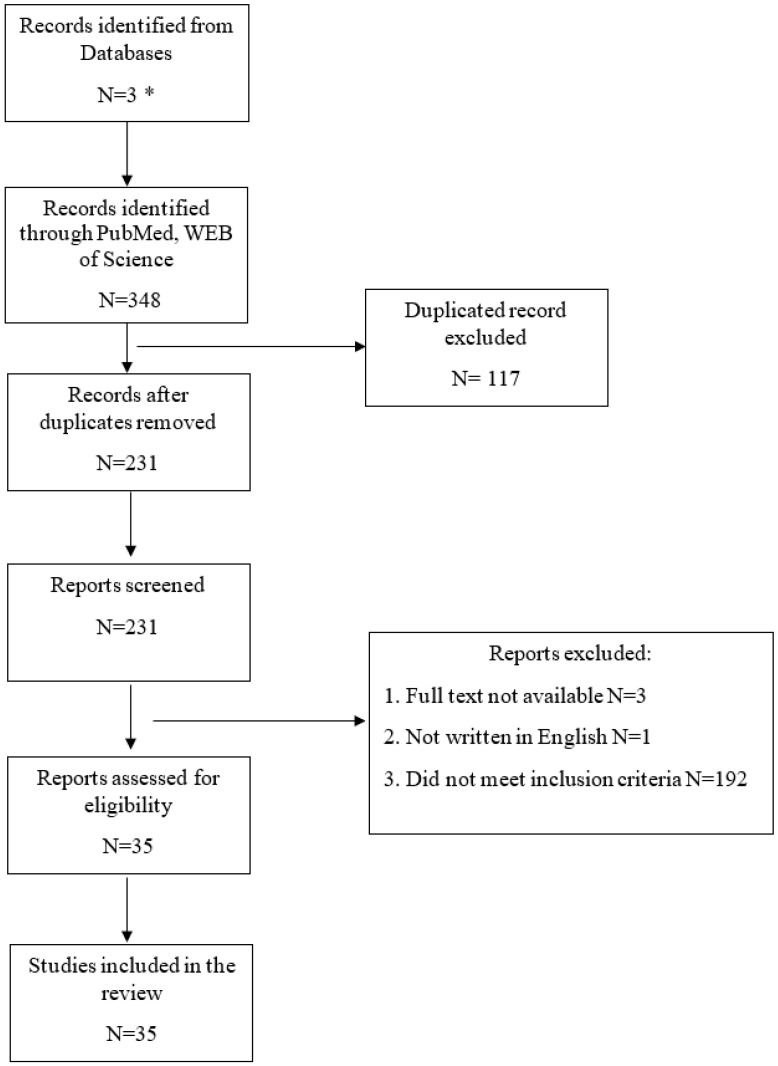
Studies of the last 5 years included in this review—PRISMA 2020 [25] flow diagram.* Web of Science, EMBASE, PubMed/Medline [https://pubmed.ncbi.nlm.nih.gov/?term=%28%22chronic+kidney+disease%22%5BAll+Fields%5D%29%29+AND+%28children%29%29+AND+%28new+markers%29%29+AND+%28blood%29%29+OR+%28%22urine+0%22%5BAll+Fields%5D%29&sort=&filter=simsearch3.fft&filter=datesearch.y_5 accessed on 6 January 2023].

Three hundred and forty-eight records were found, but only thirty-five were under PRISMA’s guidelines. The excluded studies were duplications or did not meet inclusion criteria; for instance, there was no clear diagnosis of CKD or condition leading to kidney dysfunction; research was performed on animals; study groups were newborns or adults; markers were not proteins; were common, such as creatinine or proteinuria, not novel or obtained in material other than blood or urine. There were also 14 case reports excluded; 2 were not available as full-size text, and 1 was not in English (Figure 1).

### 2.2. Data Analysis

We designed a search protocol to include as much research on new potential kidney biomarkers as possible but only in the pediatric population with a clear CKD diagnosis or condition leading to the disease. The methodology of these studies may vary as the authors used different ways of assessing protein markers from the ELISA system when single proteins were involved to proteomics detecting whole panels of molecules. Moreover, examined populations were diverse both in terms of the causes of CKD and the number of participants included. Therefore, direct statistical analysis of these data was not conducted, and we only presented obtained values of the examined molecules and described associations between examined markers and other CKD features (Table 1).

## 3. Results

The review of the existing literature on protein kidney biomarkers of CKD in children brought a broad spectrum of molecules detected in the urine and blood of affected subjects. The most studied are listed and discussed in the next section. Some molecules may detect the injury of kidney structures, while others predict the progression of the disease. Furthermore, few seem to combine these two roles. There are also markers that might even become a therapeutical target. As there is a wide diversity of these proteins, we decided to divide them according to the role they play in the process of progression of CKD and add commentary concerning their usefulness in the diagnosis of CKD.

Summarized results of studies from the last five years on protein CKD markers in children are organized in Table 1.

## 4. Discussion

### 4.1. Biomarkers of Tubular Injury

#### 4.1.1. Neutrophil Gelatinase Associated Lipocalin (NGAL)

NGAL, also known as lipocalin-2, is a 25 kDa protein produced by activated neutrophils as an innate antibacterial factor. However, NGAL seems to have more complex activities than the bacteriostatic effect. It can be found in the proximal and distant tubular cells of the kidney and, to some extent, is produced by epithelial cells of other organs, including the lungs, trachea, prostate or intestine [57]. It is freely filtered in glomeruli and, in normal conditions, undergoes complete reabsorption in the proximal tubule through the megalin, which is an endocytic receptor. Its expression is up-regulated mainly in tubules 2–4 h following acute nephrotoxic and ischaemic kidney injury; therefore, its role as an early marker was especially important in acute kidney damage [58,59,60]. Urinary (uNGAL) as well as serum (sNGAL) increase is also observed in patients with CKD. Older pediatric studies showed the usefulness of uNGAL as a predictor of the severity of lupus glomerulonephritis and hemolytic uremic syndrome [61,62]; however, most research was based on adult populations. According to Smith et al., who assessed baseline uNGAL in adult patients with CKD stage 3 or 4, it was associated with faster progression of CKD within 1 year or end-stage renal disease (ESRD) within 2 years [63]. Similar conclusions were drawn by Liu et al. [64]. There were suggestions from pre- and clinical studies that uNGAL decreases after implementing CKD treatment [65,66]. Multiple recent studies on NGAL in children with CKD of various causes mostly confirm its important role as a diagnostic tool and predictor of disease progression. NGAL seems to be one of the most promising kidney biomarkers, especially in all clinical situations related to the risk of AKI. A recent study by Greenberg et al. [26] examined urinary NGAL and other markers (IL-18, KIM-1, MCP-1, YKL-40) in a cohort of children 5 years after cardiac surgery divided into two groups (AKI vs. no AKI), revealed that uNGAL and uIL-18 were rising within 24 h post cardiopulmonary bypass with a drop in the next days. During the 5 years of follow-up, biomarkers concentrations were not significantly different in a cohort of children with AKI vs. no AKI, as well as with and without CKD or hypertension. However, children with CKD or hypertension had a higher prevalence of abnormally high uNGAL. This finding, although interpreted with caution due to the limitations of the study (relatively small number of CKD children, one cause of CKD, short period of follow-up), may indicate that NGAL might be used for screen and predicting the progression of renal disease. There are a few more studies on NGAL in CKD in children, and most of them have promising results (Table 1).

#### 4.1.2. Interleukin 18 (IL-18)

Urinary IL-18 (uIL-18) is an inflammatory cytokine produced by macrophages and proximal tubular cells as an answer to ongoing injury and subsequent inflammatory reactions involving tubules [67]. It takes part in the ischemia-reperfusion process, infections and autoimmune disorders, and similarly to uNGAL, it is a marker of an early AKI as it increases after 6–24 h post-initiating factor [68]. There are studies indicating that it could also be useful in CKD patients. In a research performed by Zubowska et al. on 85 pediatric oncology patients, IL-18 in urine was assessed to identify the group endangered with subclinical renal dysfunction [69]. After 4.6 years of observation, patients after nephrectomy and chemotherapy had significantly higher levels of uIL-18. Other studies concerning IL-18 in CKD were conducted mainly on adult populations [70,71,72]. For instance, a cohort of 153 transplant recipients showed that higher uIL-18 on the first day of post-transplantation was a predictor of faster deterioration of graft function a year post-transplant [70]. The results of the most recent research (Table 1) on uIL-18 as a predictor of CKD in children are quite conflicting and need further observations on larger and homogenous groups of subjects.

#### 4.1.3. Kidney Injury Molecule—1 (KIM-1)

KIM-1 is a transmembrane protein produced by proximal tubules and undetectable in a healthy kidney but is released and present in plasma and urine after renal injury [73]. Moreover, its expression was confirmed in lymphocytes T to stimulate lymphocyte proliferation and production of other cytokines [73]. Obviously, KIM-1 is detected after 12–24 h post-acute kidney injury (AKI) [74,75], but in several studies in children with different chronic kidney conditions such as diabetic kidney- or steroid-resistant nephrotic syndrome (SRNS), urinary KIM-1 (uKIM-1) was also markedly increased [76,77]. Bienias et al. showed that young patients with SRNS had higher levels of uKIM-1 compared to steroid-dependent patients [76]. Similarly, in the Chronic Kidney Disease in Children (CKiD) cohort, the largest longitudinal study of children with CKD from 54 North American centers, plasma KIM-1 was higher in patients with glomerular etiology of kidney disorder than those with nonglomerular [39,51]. Furthermore, it was independently related to CKD progression in an overall cohort of CKiD (aHR 4.29; 95% CI, 2.49 to 7.38) [39,51]. UKIM-1 was also associated with an increased risk of CKD progression when comparing the highest to the lowest quartile (aHR 3.03; 95% CI, 1.92 to 4,76), and it seemed to have a similar effect with urinary epidermal growth factor (uEGF). When summarising one of the most reliable studies on KIM-1 in children with CKD, both plasma and urinary KIM-1 were associated with the progression of CKD; however, urinary KIM-1, rather than plasma KIM-1, is statistically a better predictor of CKD progression as it improved three independent clinical risk prediction metrics: c-statistics, integrated discrimination improvement and continuous net reclassification improvement [39,51]. Other results of the recent studies, in most cases, confirm the important role of KIM-1 as a marker of CKD in children (Table 1).

#### 4.1.4. Epidermal Growth Factor (EGF)

In humans, EGF is a protein expressed in the thick ascending limb of Henle’s loop and distal tubules [78]. It interacts with the EGF receptor and promotes the regeneration of tubular cells after injury. We can confirm that EGF is a marker of tubular health and plays a protective role in the nephron. Urinary EGF (uEGF) is a good candidate biomarker of CKD as it is highly specific for kidney tissue [78]. In the experimental study in rats with induced bilateral occlusion of the renal arteries, Norman et al. observed that treatment with exogenous EGF attenuated the rise in serum creatinine [79]. There are studies in adults indicating that adding uEGF to conventional markers of CKD (eGFR, albuminuria) improved the prediction of the progression of the disease [80]. One of the most reliable studies in children in the CKiD cohort revealed that patients with lower baseline uEGF developed progression of CKD. Children who had uEGF in the lowest quartile had a 7-fold higher risk of progression of CKD compared to patients with uEGF in the highest quartile (aHR 7.14, 95%CI, 3.45 to 20.0) [74]. These findings are consistent with newer observations on the 4C pediatric cohort, designed to assess cardiovascular comorbidity in 704 children with CKD (GFR 10–60 mL/min/1.73 m^2^) in 14 European countries. In this study, higher uEGF was independently related to decreased risk of progression of CKD [31]. It seems that uEGF, as a highly specific stimulator of tubular regeneration, may serve as a good biomarker of CKD progression; furthermore, it might become a potential target for implementing future therapies. Findings of the most recent studies on EGF in CKD in children are presented in Table 1.

#### 4.1.5. α1-Microglobulin

α1-microglobulin is a small protein primarily produced by the liver as a free radicals scavenger, filtered by the glomerulus and reabsorbed by proximal tubular cells. It is present in urine after proximal tubular dysfunction [81]. Increased urinary levels of α1-microglobulin were found in adults with a progression of CKD and cardiovascular disease [82,83]. In the pediatric CKiD cohort, it was higher in patients with progression of CKD in comparison to those with stable kidney disease, but this was shown only in the unadjusted model [51]. This observation does not prove the significance of α1-microglobulin as a reliable biomarker of CKD in children, and further research in this field is necessary.

### 4.2. Biomarkers of Inflammation

#### 4.2.1. Tumor Necrosis Receptor 1 and 2 (TNFR1 and TNFR2)

Tumor necrosis factor α (TNFα) is a multifunctional cytokine that plays a pivotal role in the process of inflammation. It regulates cell proliferation, differentiation and apoptotic death [84]. TNFα interacts with two membrane receptors, TNFR1 and TNFR2. TNFR1 is primarily found in glomeruli and peritubular endothelium, while TNFR2 can be transcriptionally induced in the kidney but is mostly expressed in lymphocytes. Stimulation of TNF receptors increases macrophage infiltration in the interstitium and interstitial fibrosis [84,85]. Animal models of obstructive uropathy showed that TNFR2 was increased in affected animals, and TNFR 2 deficient mice had significantly less tubulointerstitial fibrosis [86]. There are several studies in adults with different causes of CKD, including diabetic nephropathy, obstructive nephropathy, lupus or ANCA-induced nephritis, and all of them showed these receptors are predictors of the progression of CKD [86,87,88]. Serum TNFR1 was higher in children with reflux nephropathy versus healthy controls [89]. A relatively new study from Brazil, although performed on a small patient group of 34 children with CKD, showed higher blood levels of TNFR1 and TNFR2 when compared to healthy controls. Both receptors inversely correlated with eGFR (TNFR1 r = −0.853 and TNFR2 r = −0.729; *p* < 0.01 for both) [90]. In the recent study of Greenberg et al. on the CKiD cohort, plasma TNFR1 and TNFR2 were strongly associated with the progression of CKD [39] (Table 1).

#### 4.2.2. Blood Soluble Urokinase-Type Plasminogen Activator Receptor (suPAR)

UPAR is a membrane receptor protein expressed in podocytes, regulating their migration, adhesion and apoptosis. It is also found in endothelium and immature myeloid cells [91]. Activated uPAR is cleaved, and the soluble form, suPAR, is present in the blood [92]. First investigations on suPAR concerned FSGS. In the study by Wei and al. on 70 participants with a histopathologic diagnosis of FSGS (children and adults), a decrease in suPAR blood concentration was related to a reduction in proteinuria and achieving remission of nephrotic syndrome [93]. SuPAR was evaluated in the two cohorts of CKD children: in the ESCAPE study (originally designed for assessment of blood pressure control and use of angiotensin-converting-enzyme inhibitors on CKD progression in pediatric patients) and the 4C trial (The Cardiovascular Comorbidity in Children with CKD). The results showed that in a group of patients with CKD and GFR >40 mL/min/1.73 m^2^, log-transformed serum suPAR concentrations were related to a higher risk of CKD progression (HR 5.12; 95% CI, 1.56 to 16.7; *p* < 0 01) [94]. In the CKiD pediatric cohort, two researchers assessed suPAR blood concentration using different methods and produced distinctive results. Widemann et al. used enzyme-linked immunoabsorbent assay (ELISA) and observed in regression models with adjustment for eGFR, proteinuria or other demographic variables that patients with plasma suPAR in the highest quartile had a 33% faster progression of CKD than individuals with suPAR in the lowest quartile [40]. Greenberg et al. measured plasma suPAR with electrochemiluminescence multiplex assay with the Meso Scale Discovery platform. They showed that suPAR was a predictor of CKD progression; however, after adjustment for other variables, the significance of this relation was lost [39] (Table 1). It should be pointed out that validation and use of approved, similar tests are essential to achieve reliable and repeatable results of the concentrations of the biomarkers.

#### 4.2.3. Monocyte Chemoattractant Protein-1 (MCP-1)

MCP-1 is a chemotactic factor for monocytes promoting their transformation into macrophages. It activates memory T lymphocytes, NK cells and basophils [95]. It is found in podocytes, endothelial and mesangial cells, and mononuclears, especially in the presence of inflammation [95]. Lupus nephritis severity correlated with urinary MCP-1 (uMCP-1) levels in pediatric patients [96]. UMCP-1 was higher in children with CKD compared to healthy controls. Furthermore, the glomerular disorder was related to even higher urinary levels of MCP-1 [97]. In the CKiD cohort, MCP-1 blood concentration was not related to CKD progression, opposite to its urinary levels [39] (Table 1). After adjustment for eGFR, hypertension and proteinuria, children with uMCP-1 in the fourth quartile had a significantly higher risk of CKD progression in comparison with those having uMCP-1 in the lowest quartile. This relation was evident in patients with autosomal recessive polycystic kidney disease (ARPKD) [51]. It is worth noticing that uMCP-1 is highly correlated with uKIM-1 (r = 0.7, *p* < 0.01) [51]. Other results of recent studies on MCP-1 in pediatric CKD are included in Table 1.

#### 4.2.4. Chitinase-3-like Protein 1 (YKL-40)

YKL-40 participates in tubular epithelium repair after ischemia-reperfusion injury [98]. There are not many studies assessing YKL-40 in kidney diseases in children, and those reviewed do not give promising results (Table 1).

### 4.3. Biomarkers of Fibrosis

#### 4.3.1. Blood Transforming Growth Factor β1 (TGF-β1)

TGF-β1 is a pro-fibrotic growth factor produced by tubular epithelial cells, fibroblasts and different inflammatory cells in the condition of persistent inflammation with irreversible tissue damage. Few studies on CKD in adults confirm increasing urinary levels of TGF-β1 with the progress of CKD [99,100]. In children with nephrotic syndrome, urinary levels of TGF-β1 were significantly higher when the etiology was FSGS compared to minimal change disease (MCD) [101]. Another pediatric study revealed higher urinary TGF-β1 in individuals with obstructive than non-obstructive uropathy [102]; however, the results of previous research do not indicate its great value as a predictor of CKD (Table 1).

#### 4.3.2. Blood Bone Morphogenic Protein-7 (BMP-7)

BMP-7 is an antagonist of TGF-β1 and prevents inflammation and subsequent fibrosis. In the study by Musial et al., children with CKD had higher concentrations of BMP-7 when compared to healthy individuals [103]. Some trials show that BMP-7 could be used in the therapy of kidney diseases as recombinant BMP-7 infusions used in the pre-clinical phase decreased fibrosis and inflammation occurring in obstructive uropathy [104] or slowed GFR decline in mice with lupus nephropathy [105]. However, we lack studies on bigger populations of children with CKD.

#### 4.3.3. Blood Matrix Metalloproteinase-2 and 9 (MMP-2, MMP-9)

Matrix metalloproteinase (proteases) family plays an important role in the degradation of the extracellular matrix. This effect is also crucial in the development and progression of CKD. MMP-2 and MMP-9 belong to gelatinases and are produced under normal conditions by the mesangial cells and tubular epithelial cells in the human kidney but at low levels [68]. However, in the process of renal fibrosis, their upregulation is very rapid due to the interactions of multiple signaling pathways and further abnormal activation [106]. MMP-2 and 9 interact with TNFs and MCP-1 in the development of CKD. MMP-2 and 9 can be detected both in blood and urine. CKD children had significantly higher plasma MMP-9 concentrations than healthy participants [107]. Similarly, pediatric patients with FSGS showed higher urinary MMP-9 in comparison with children with MCD or the control group [108]. In the recent study by Stabouli et al., MMP-2 rather than MMP-9 correlated with the progression of CKD in children (Table 1) [102].

#### 4.3.4. Urinary procollagen III N-terminal peptide (PIIINP)

PIIINP, as a byproduct of collagen 3 deposition, is another marker of fibrosis. In a study on children with ureter obstruction, urinary PIIINP was related to worsening kidney blockage [109]. It also correlated with more intense interstitial fibrosis in the biopsies of the kidney with CKD [109] and was a marker of CKD progression in renal transplant recipients [110]. The study by Taranta-Janusz et al. showed that children with solitary functioning kidneys (SFK) had significantly higher urinary PIIINP than healthy controls [33].

### 4.4. Miscellaneous

Several studies in our research included markers that do not fit the preceding categories. Fibroblast growth factor 23 (FGF23) is somewhere between the “old-fashioned” kidney function indicators and newer markers. FGF23 is a phosphaturic factor as it inhibits both phosphate reabsorption in the kidney and vitamin D activation. It is crucial for the development of secondary parathyroidism in the course of CKD [111] and rises in the early stage of CKD [112]. In the CKiD pediatric cohort, Portale et al. revealed that FGF23 was associated with CKD progression, even after adjustment for the age of the patients, mineral metabolites and other CKD-specific factors [112]. Another protein that might be a potential marker of kidney function is liver-type fatty acid-binding protein (L-FABP). It is a cytoplasmic protein existing mainly in adipose tissues and is expressed not only in the liver or pancreas but also in the kidney [113]. L-FABP presented in the proximal tubules can likely be used for the detection and monitoring of AKI or CKD [114]. Assessing urinary L-FABP seems to be less susceptible to being influenced by other conditions such as heart failure or metabolic syndrome. In the recent study by Lipiec et al., children after HUS had significantly higher urinary and serum L-FABP values when compared to healthy controls (results are shown in Table 1) [29].

The next protein that should be mentioned is retinol-binding protein (RBP), a transporter molecule for vitamins synthesized in the liver [115]. RBP is filtered by the glomerulus, reabsorbed and degraded in proximal tubules; therefore, urinary RBP can be used for monitoring renal tubular dysfunction [68]. Together with vitamin D-binding protein (VDBP) and heat shock proteins (hsp27 and HSF1) are markers of tubular function and apoptosis that accompany CKD from the very beginning. In the study by Musiał et al. [25], urinary fractional excretion (FE) of RBP4, VDBP, hsp27 and HSF1 was assessed in 70 children with CKD stages 1–5 treated conservatively and 12 age-matched healthy peers. It appeared that FE values of all parameters exceeded 1% in CKD st. 2 and raised significantly versus the control group: in stage 2, it was RBP4 and HSF1; in stage 3, it was VDBP; and in stage 4, it was Hsp27 (Table 1) [25].

A few other molecules were investigated as potential kidney biomarkers of CKD in children: clusterin, osteopontin, N-Acetyl-β-d-amino Glycosidase (NAG), symmetric and asymmetric dimethylarginine (SDMA, ADMA), or Trefoil Factor3 (TFF3) and results of recent studies were presented in Table 1. None of them has an unequivocal position of a useful diagnostic indicator, and further research on this field is necessary.

In this review, we concentrated on protein markers that may serve as a diagnostic tool for CKD. Nevertheless, it is worth noticing that also metabolomic profiling may bring us the discovery of potential metabolite associations with CKD in children. In the study of Denburg et al. [54], the authors evaluated metabolomics quantification of plasma samples from 645 children (median age 12 years) with chronic kidney disease (CKiD cohort participants). Metabolites were standardized and logarithmically transformed. The main issue was examining the association between 825 nondrug metabolites and progression to the composite outcome of KRT or 50% decline of eGFR adjusting for age, sex, race, body mass index, hypertension, glomerular vs. nonglomerular diagnosis, proteinuria, and baseline eGFR. The median time of follow-up was 4.8 years. It was shown that among participants with baseline eGFR ≥ 60 mL/min/1.73 m^2^, two-fold higher levels of seven metabolites were significantly associated with higher hazards of KRT/halving of eGFR events: three of purine or pyrimidine metabolism (N6-carbamoylthreonyladenosine, dihydrouridine, pseudouridine), two amino acids (C-glycosyltryptophan, lanthionine), the tricarboxylic acid cycle intermediate 2-methylcitrate/homocitrate and gulonate. Among those with baseline eGFR < 60 mL/min/1.73 m^2^, a higher level of tetrahydrocortisol sulfate was associated with a lower risk of progression.

## 5. Conclusions

In this review, we provided insight into the most recent research on protein kidney markers with the potential for becoming a useful diagnostic indicator of CKD or even a target for future therapies. Despite there being so many studies in this field, we still do not have consistent conclusions about which molecules have the best utility as markers of CKD. One of the most promising seems to be NGAL; however, not when it is alone. Combination with EGF, which is highly specific for kidney tissue, might improve the identification of the risk of CKD progression. Nevertheless, “old type” markers such as creatinine, albuminuria or proteinuria, despite their limitations, are still used in the pediatric CKD population, and we cannot underestimate their importance in clinical practice [116]. There is a great need for molecules that react early in kidney deterioration and are cheap, easy to assess and independent from other factors. It would be perfect to use them together with those “old and tried” markers. Nevertheless, more organized research on larger pediatric populations with CKD and with the use of validated and uniform methodology should be conducted.

## Figures and Tables

**Table 1:** Results of the studies from the last five years on new protein kidney biomarkers in CKD in children.

Year	Author	Study Design	Results
NGAL			
2018	Greenberg et al. [26]	- Prospective cohort of children 5 years after cardiac surgery, with a 49% rate of AKI because of surgery with cardiopulmonary bypass and with potential risk for long-term CKD. - From the total of 305 participants, 110 took part in the 5-year follow-up. - Age of participants during cardiac surgery: 1 month—18 years old. - measurement of urinary: NGAL, IL-18, KIM-1, MCP-1, YKL-40. - Biomarkers levels were compared between patients who had postoperative AKI and those without this complication. - Cross-sectional analysis between the biomarkers and hypertension or CKD was performed.	- None of the biomarkers was associated with CKD or hypertension in 5 years follow-up. - Values of assessed markers after 5 years of follow-up in the overall group of participants were as follows, with no significant differences between patients with or without postoperative AKI (expressed as median with interquartile range): uNGAL 4.4 (2.1–10.2) ng/mL; uIL-18 14.7 (8.6–23.4) pg/mL; uKIM-1 250.6 (157.6–568.5) pg/mL; uMCP-1 109.9 (46.4–169.4) pg/mL; uYKL-40 336 (157.1–615.1)pg/mL. - There was no significant difference in eGFR between patients with or without postoperative AKI, and overall was 113 (103–126) mL/min/1.73 m^2^; however, CKD and hypertension were present in 18 (17%) and 20 (18%) children, respectively. - Only uNGAL was elevated in children with CKD and with hypertension after 5 years of observation.
2018	Bienias et al. [27]	- 45 children with congenital unilateral hydronephrosis (HN) due to ureteropelvic junction obstruction and 21 healthy controls - Assessment of new kidney biomarkers including urinary: glutathione S-transferases (GST) alpha-GST, pi-GST, NGAL, KIM-1 (expressed as ratio to creatinine) and serum sNGAL.	- Patients with the highest grade of (HN) showed significantly increased values of all urinary and serum biomarkers, whereas those with the lowest grade HN showed only significant elevation of sNGAL; values below, patients with the highest grade of HN (n = 25) vs. control group (expressed as median with interquartile range). Urinary: alpha-GST (ng/mg cr.) 4.51 (0.54–17.3) vs. 1.11 (0.26–3.5); pi–GST (ng/mg cr.) 30.4 (17.5–24.9) vs. 14.6 (7.4–28.5); NGAL (ng/mg cr.) 1.73 (0.17–10) vs. 0.83 (0.04–9.5); KIM–1 (ng/mg cr.) 2.4 (0.2–5.1) vs. 0.28(0.06–1.06). Serum: NGAL (ng/mL) 59.9 (45.2–85.3) vs. 4.8 (2.1–10.4). - uNGAL positively correlated with a percentage loss of relative function of an obstructed kidney in renal scintigraphy (r = 0.5, *p* < 0.05). - ROC curve analysis showed the best diagnostic profile for uNGAL/cr and alpha-GST/cr in the detection of obstructive nephropathy.
2018	Wu et al. [28]	- 60 pediatric cases with SLE and LN, 29 without LN and 22 healthy controls. - Assessment of urinary biomarkers: b2-microglobulin, cystatin C, KIM-1, MCP-1 clusterin, calbindin, IL-18, NGAL, TFF3, osteopontin and glutathione S-transferase for their ability to predict renal histopathologic findings and progression to end-stage kidney disease.	- Urinary albumin and clusterin were elevated in patients with tubulointerstitial lesions (*p* = 0.035 and 0.048, respectively). - Urinary clusterin performed best at predicting end-stage kidney disease with cutoff of 0.61 × 10^4^ (AUC 0.804, *p* = 0.002).
2018	Musiał et al. [25]	- 70 children with CKD stages 1–5 treated conservatively, and 12 age-matched healthy peers. - Assessment of the usefulness of fractional excretion (FE) of vitamin D-binding protein (VDBP), retinol-binding protein (RBP4) and heat shock proteins (hsp).	- FE values of all parameters exceeded 1% in CKD st. 2 and raised significantly versus control group: in stage 2—RBP4 and HSF1; stage 3—VDBP; stage 4—Hsp27.
2018	Lipiec et al. [29]	- 29 children with a history of HUS endangered with risk of CKD compared to healthy peers. - Assessment of L-FABP and interleukin 6 (IL-6) in serum and urine.	- Children after HUS had significantly higher L-FABP and IL-6 in both serum and urine when compared to healthy peers Values below: group after HUS vs. healthy peers (expressed as a mean and SD): sIL-6 (ng/mL) 79.96 ± 26.68 vs. 7.34 ± 1.43; uIL-6 (ng/mL) 97.64 ± 15.46 vs. 36.87 ± 12.01; sL-FABP (ng/mL) 72.49 ± 18.53 vs. 2.65 ± 0.75; uL-FABP (ng/mL) 11.54 ± 4.32 vs. 2.76 ± 0.63.
2019	Bartoli F. et al. [30]	- 80 children with CAKUT (40 hypodysplasia, 22 agenesic, 10 multicystic, 8 nephrectomy) after extensive urological and nephrological workups below 14 years old and without recent urinary tract infection or hypertension and compared to 30 healthy controls. - Evaluation urinary levels of MCP-1, EGF, b2-microglobulin, FAS-ligand. - Assessment of urinary ratios uEGF/uMCP-1 (regenerative versus inflammatory response) and uEGF/ub2-microglobulin (regeneration versus tubular damage).	- uEGF values (pg/mL, mean, SD) were significantly lower in controls (515 ± 168) and nephrectomy children (408 ± 201) in comparison to multidysplastic (794 ± 243), hypodysplastic (754 ± 435) and agenesic (628 ± 252) patients. - Urinary levels of MCP-1 (pg/mL, mean, SD) were highest in multicystic patients (3.3 ± 1.3), but hypodysplastic, agenesic and multicystic participants also had uMCP-1 up-regulated in comparison to controls (2.2 ± 1.6). - Both ratios, uEGF/uMCP-1 and u EGF/ub2-microglobulin, were downregulated in all CAKUT children when compared to healthy participants.
2019	Azukaitis et al. [31]	- Children with CKD from 4C study (Cardiovascular Comorbidity in Children with CKD) with baseline eGFR 10–60 mL/min/1.73 m^2^. - CAKUT were the most common cause of CKD, while glomerular disease accounted for less than 10% of cases. - Urinary EGF (uEGF) measured 6 months after enrolment.	- In a Cox proportional hazards model, higher uEGF/cr was associated with a decreased risk of CKD progression (HR 0.76; 95% CI 0.69–0.84).
2019	Musiał et al. [32]	- 41 CKD children (19 patients on haemodialysis, 22 children on automated peritoneal dialysis) and 23 healthy controls. - Assessment of serum concentration of MCP-1, MCSF and neopterin.	- serum MCP-1, MCSF and neopterin were significantly elevated in all patients versus controls, and the highest values were obtained in hemodialyzed children. - Single hemodialysis sessions lowered the concentrations of all markers; however, they rose before the next procedure.
2019	Taranta-Janusz et al. [33]	- 98 children with solitary functioning kidney (SFK) with a median age of 8 years compared to matched 54 healthy peers. - Assessment of PIIINP and beta-catenin in urine.	- uPIIINP in SFK children was statistically higher compared to controls. - No difference in median urinary beta-catenin between the two groups.
2020	Nickavar et al. [34]	- 32 children with primary vesicoureteral reflux (mean age 36.84 ± 28.16 months) and 37 healthy peers (mean age 32.32 ± 29.08 months) were evaluated. - Comparison of uNGAL and uNGAL/cr between children with and without VUR. - Kidney parenchymal function assessed using DMSA scans.	- Mean uNGAL and uNGAL/cr were higher in patients with VUR. - The optimal predicting cutoff value for uNGAL/cr with the highest sensitivity and specificity was 0.88 ng/mg (sensitivity 84%, specificity 81%). - uNGAL/cr significantly increased in patients with decreased parenchymal function according to DMSA scans (16.89 ± 32.34 vs. 3.74 ± 10.8 ng/mg, *p* = 0.041).
2020	Staub et al. [35]	- A group of 51 former preterm infants, aged 10–15 years old, were assessed for blood pressure and kidney markers measured in serum and urine (creatinine, NGAL, uromodulin) and only serum (cystatin C, beta-2-microglobulin, beta trace protein) and compared to 82 term-born controls.	- Serum: creatinine and NGAL were significantly higher in the preterm group. - No association between the term of birth and other biomarkers was found.
2020	Latoch et al. [36]	- 60 patients previously treated for acute lymphoblastic leukemia (ALL) compared to 53 healthy peers. - Median time after cessation of treatment was 6.55 years, with a median age of 12 years. - Assessment of uNGAL and uKIM-1 and expressed as a ratio to creatinine (uNGAL/cr, uKIM-1/cr) in the study group with consideration of time after the end of treatment, eGFR and cumulative doses of methotrexate and cyclophosphamide.	- Median levels of both uNGAL and uNGAL/cr were significantly higher in ALL survivors than healthy children (uNGAL 3.98; 1.48–11.45 vs. 0.004; 0.001–0.005 ng/mL, *p* < 0.0001; uNGAL/cr 31.37, 14.23–84.67 vs. 0.004, 0.002–0.007 mg/mg, *p* < 0.0001; data given as median with interquartile range). - uNGAL seemed to be the best predictor of decreased eGFR (AUC = 0.67). - uKIM-1/cr was significantly higher in ALL survivors than in healthy children (uKIM-1/cr 6.16; 3.29–9.98 vs. 0.93, 0.44–1.48 ng/mg, *p* < 0.0001; data given as median with interquartile range). - ALL survivors had statistically higher uKIM-1 and eGFR 5 years after the end of treatment when compared to those with an observation period of fewer than 5 years. - Cumulative doses of methotrexate and cyclophosphamide did not predict the values of uNGAL and uKIM-1.
2020	Gul et al. [37]	- 50 obese and 26 overweight adolescents aged 10–16 years old compared to 26 normal body weight children in the control group. - Assessment of uNGAL and uKIM-1 in examined children and expressed as a ratio to creatinine.	- No significant differences in uNGAL and uNGAL/cr were found between obese, overweight and normal body weight participants. - uNGAL was higher in overweight or obese children with LDL dyslipidemia as compared to those with LDL within reference values (64.12, 30.98–114.32 vs. 39.51, 25.59–56.37 ng/mL; *p* = 0.024, data presented as median, Q1–Q3). - There was a correlation between insulin levels (insulin resistance) and uNGAL/cr in overweight participants (r = 0.515, *p* = 0.008) but not in the obese group. - No significant differences in uKIM-1 were found between obese, overweight and normal body weight participants.
2020	Nickavar et al. [34]	- Comparison of uNGAL between children with and without VUR. - Kidney parenchymal function assessed using DMSA scans.	- Mean uNGAL and uNGAL/cr were higher in patients with VUR. - uNGAL/cr significantly increased in patients with decreased parenchymal function according to DMSA scans
2020	McLeod et al. [38]	- 22 children with obstructive uropathy were identified in the CKiD cohort receiving KRT and compared to 22 KRT-free controls. - Measurement of urinary and plasma NGAL, IL-18 and L-FAB at enrolment and annually during 5 years of follow-up and comparison between cases and controls.	- No difference between examined biomarkers between KRT children and controls at baseline. - Mean pNGAL and uL-FABP/cr increased throughout the study period in cases (15.38 ng/mL per year and 0.2 ng/mL per mg/dl per year, respectively, *p* = 0.01 for both) while remaining stable in controls.
2020	Greenberg et al. [39]	- 651 participants from the CKiD cohort with eGFR 30–90 mL/min/1.73 m^2^ with its further assessment annually. - 195 had a glomerular and 456 nonglomerular cause of CKD. - Median age of study participants was 11 years, with the diagnosis of CKD at a median of 8.2 years, and the median follow-up time was 5.7 years. - Assessment of kidney biomarkers in serum: KIM-1, YKL-40, MCP-1, suPAR and TNFR-1/TNFR-2 in relation to eGFR decline in subgroups (glomerular versus nonglomerular cause of CKD) twice: a) baseline (5 months after study enrolment); b) in the primary endpoint (CKD progression defined as 50% decline in EGFR or ESKD).	- All biomarkers were inversely correlated with eGFR, with the strongest relationship between eGFR and TNFR-1 (r = −0.74) - After multivariable adjustment, children with a plasma KIM-1, TNFR-1 and TNFR-2 concentration in the highest quartile were at significantly higher risk of CKD progression compared with children with a concentration of the respective marker in the lowest quartile (a 4-fold higher risk for KIM-1 and TNFR-1 and a 2-fold higher risk for TNFR-2). - Plasma MCP-1, suPAR and YKL-40 were not independently associated with CKD progression.
2020	Weidemann et al. [40]	- 565 participants (age 1–16 years) enrolled from the CKiD cohort. - Assessment of plasma suPAR concentration and categorized by quartiles, measured at study entry and after a 6-month follow-up interval. - Outcome was CKD progression, defined as the need for KRT or more than a 50% decline in eGFR.	- Participants in the highest quartile of plasma suPAR concentration had 54% faster progression of CKD in comparison to those in the lowest quartile. - Plasma suPAR levels showed little change over 6 months.
2020	Musiał et al. [41]	- 70 children with conservatively treated CKD stages 1–5 and 12 healthy controls. - Assessment of serum and urine concentration of MCP-1, MCSF, TIMP-2 and survivin.	- Serum concentrations of all parameters were significantly elevated at CKD stage 1 compared to controls - Urinary MCP-1 and MCSF (stages 1–2) rose earlier than TIMP-2 or survivin
2020	Stabouli et al. [42]	- 33 CKD participants, 18 T1D patients, 24 healthy controls. - Assessment of MMP-2 and MMP-9 between studied groups. - Measurements of office BP, pulse wave analysis and carotid–femoral pulse wave velocity.	- MMP-2 values were higher in the CKD compared to diabetes patients and healthy participants. - MMP-9 values did not differ between these groups. - Only MMP-2 correlated positively with creatinine in CKD patients and negatively in diabetic participants. - MMP-2 was associated with arterial stiffness indicators in the presence of hypertension.
2021	Anand et al. [43]	- 50 children with CAKUT, aged <14 years old, were divided into group 1 (without CKD) or group 2 (with CKD) compared to healthy peers. - Measurement of NGAL; trefoil family factors (TFF) 1, 3; and albuminuria in the urine at the beginning and after 1 year of follow-up. Kidney function was assessed using DTPA, DMSA scans and GFR at baseline and after 1 year of observation (assessed using Schwartz formula).	- Median concentrations of uNGAL (281.2 mcg/g cr.), TFF 1 (44.5 mcg/gcr.) and TFF 3 (176.5 mcg/g cr.) were significantly higher in CAKUT patients in comparison to controls (*p* < 0.05). - Children with progressive deterioration of kidney function (group 2) had higher uNGAL than those from group 1 (without CKD progression). - TFF 3 was found to have the highest AUC (0.919) on the ROC curve for predicting progressive kidney functional deterioration.
2021	Leiber et al. [44]	- Cross-sectional study of 210 children living in a MeN endemic region of Nicaragua. - Evaluation of urinary kidney biomarkers: NGAL, KIM-1, IL-18, MCP-1, YKL-40 and its association with eGFR. - Comparison of the levels of the above markers between the study group and healthy children from other countries.	- Median uNGAL, uIL-18 and uKIM-1 were significantly higher in the study group. - A one-year increase in age was associated with a 40% increase in odds of being in the highest quartile of uNGAL. - Children with dysuria had 2.5 times the odds of having uNGAL in the highest quartile.
2021	Będzichowska et al. [45]	- 59 children with kidney disorders that had indications for renoscintigraphy were divided into two groups with renal scarring and with normal kidney pictures. - Assessment of eGFR calculated by Schwartz formula (children with glomerular hyperfiltration defined as EGFR ≥ 130 mL/min/1.73 m^2^ versus normal filtration with eGFR <130 mL/min/1.73 m^2^) and new kidney biomarkers, including serum: KIM-1, FGF-23, NAG, NGAL and uromodulin, and urinary: NGAL and uromodulin.	- Children with hyperfiltration had higher sNGAL and FGF-23. - No significant differences were found between the children with hyperfiltration and normal filtration in terms of blood levels of KIM-1, FGF-23, NAG, NGAL and uromodulin, and urinary NGAL and uromodulin.
2021	Jacobson et al. [46]	- 618 participants from the CKiD cohort (pediatric CKD patients from the US and Canada) were enrolled to assess whether exposure to environmental chemicals, such as pesticides, impacts renal function and chronic kidney disease (CKD). - Children were followed over an average of 3 years. - In serially collected urine samples over time, six nonspecific dialkyl phosphate (DAP) metabolites of pesticides were measured together with kidney biomarkers: NGAL, KIM-1. - Assessment of eGFR and proteinuria was performed annually.	- There was no relation between DAPs and uNGAL or other clinical renal outcomes: proteinuria and blood pressure. - Although DAPs were associated with lower eGFR at baseline, there was a tendency of higher eGFR over follow-up that seemed to be inconsistent. - DAPs were associated with increased KIM-1 urinary excretion over time, suggesting the presence of subclinical kidney injury.
2021	Sethi et al. [47]	- 44 children after cardiac surgery with cardiopulmonary bypass who had postoperative AKI in comparison to 49 healthy peers. - Median follow-up of 41 months. - Assessment of urinary: NGAL, IL-18, KIM-1 with adjusting for creatinine concentration.	- The cases had significantly higher uNGAL, uIL-18 and uKIM-1 on follow-up, and values remained higher after adjusting for urine creatinine, - None of the patients had proteinuria or hypertension,
2021	Ahn et al. [48]	- 55 children and adolescents with T1D and T2D were divided into two subgroups with normal and high albuminuria. - 44 healthy controls. - Evaluation of KIM-1 in urine and plasma.	- pKIM-1 concentration was significantly higher in diabetic children compared to controls. - Similarly, pKIM-1 was higher in high albuminuric than normoalbuminuric participants. - HbA1c was identified as an important risk factor for increased pKIM-1.
2021	Williams et al. [49]	- Systematic review of urine biomarkers in children with IgA vasculitis nephritis in terms of clinical and pre-clinical ability to predict the presence of nephritis and subsequent CKD. - 13 eligible studies with a total of 2446 pediatric patients: 1236 children with IgAV-N, 449 children with IgAV without nephritis and 761 healthy controls - Assessment of severity of nephritis with “old” indicators: 24-hour protein excretion in urine, protein/creatinine ratio in urine, urinary albumin concentration.	- Most promising urinary biomarkers in predicting the presence of nephritis were: KIM-1 (AUC 0.93), MCP-1 (AUC 0.83), NAG (0.76). - They appeared to correlate with disease severity
2021	Nosek et al. [50]	- 80 children with congenital or acquired SFK compared to healthy controls. - Serum TWEAK and urinary MCP-1 and RANTES were assessed in relation to kidney function (serum creatinine, eGFR, albuminuria, hypertension)	- Serum TWEAK and urinary MCP-1 and RANTES levels were higher in SFK patients
2021	Sandokij et al. [51]	- participants from the CKiD cohort, the group from the previous study of Greenberg et al. [38]. - uMCP-1 assessment of baseline (5 months after study enrolment) and in the primary endpoint (CKD progression defined as 50% decline in EGFR or ESKD). - Children with different causes of CKD (glomerular versus nonglomerular)	- In the CKiD cohort, uMCP-1 was related to CKD progression. - After adjustment for eGFR, hypertension and proteinuria, children with uMCP-1 in the fourth quartile had a significantly higher risk of CKD progression in comparison with those having uMCP-1 in the lowest quartile. - It was evident in patients with autosomal recessive polycystic kidney disease (ARPKD). - uMCP-1 was highly correlated with uKIM-1 (r = 0.7, *p* < 0.01).
2021	Turczyn et al. [52]	- 81 children with congenital obstructive nephropathy and 60 healthy controls. - Assessing the severity of renal fibrosis based on 99mTC-ethylenedicysteine scintigraphy scans: severe, moderate and borderline lesions. - Investigation of predictive value if urinary endoglin, periostin, cytokeratin-18, TGF- β1.	- Urinary: periostin, periostin/cr and cytokeratin-18 levels were higher in the study group when compared to controls. - Children with severe scars had higher urinary periostin/cr than those with borderline changes. - Periostin and cytokeratin-18 were independently related to the presence of severe and moderate scarring. - TGF- β1 demonstrated low utility for assessing renal fibrosis in children with obstructive nephropathy.
2021	Tuncay et al. [53]	- 50 patients diagnosed with pre-dialytic CKD and 30 healthy controls. -Measurement of serum: IL-8, IL-10, IL-13 and TGF- β1. - Assessment of carotid–femoral pulse wave velocity, carotid intima-media thickness and left ventricular mass index as markers of cardiovascular disease.	- Only serum IL-8 was higher in CKD patients. - No difference in levels of IL-8, IL-10, IL-13, TGF- β1 in CKD patients with and without cardiovascular disease.
2021	Denburg et al. [54]	- Metabolomics quantification of plasma samples from 645 children (median age 12 years) with chronic kidney disease (CKiD cohort participants). - Metabolites were standardized and logarithmically transformed. - Assessing the association between 825 nondrug metabolites and progression to the composite outcome of KRT or 50% decline of eGFR adjusting for age, sex, race, body mass index, hypertension, glomerular vs. nonglomerular diagnosis, proteinuria and baseline eGFR. - Median time of follow-up was 4.8 years.	- Among participants with baseline eGFR ≥ 60 mL/min/1.73 m^2^, two-fold higher levels of seven metabolites were significantly associated with higher hazards of KRT/halving of eGFR events: three of purine or pyrimidine metabolism (N6-carbamoylthreonyladenosine, dihydrouridine, pseudouridine), two amino acids (C-glycosyltryptophan, lanthionine), the tricarboxylic acid cycle intermediate 2-methylcitrate/homocitrate and gulonate. - In those with baseline eGFR < 60 mL/min/1.73 m^2^, a higher level of tetrahydrocortisol sulfate was associated with a lower risk of progression of CKD.
2022	Johnson et al. [55]	- 50 adolescents with T1D were examined and compared to 20 healthy BMI- and age-matched controls. - Assessment of the relationship between intrarenal hemodynamic function (assessed with intraglomerular pressure, efferent arteriole resistance, afferent arteriolar resistance or renal plasma flow, GFR, albumin/creatinine ratio) and kidney biomarkers in urine (NGAL, IL-18, KIM-1, YKL-40, MCP-1 and copeptin).	- YKL-40 and KIM-1 concentrations were positively associated with GFR, renal plasma flow, albumin/creatinine ratio, and intraglomerular pressure in T1D adolescents. - No significant association between NGAL, IL-18, MCP-1, and intrarenal hemodynamic function indicators was found.
2022	Guansekara et al. [56]	- School students (10–18 years old) from endemic areas are endangered with CKD compared to children from non-endemic regions. - Assessment of the utility of kidney biomarkers in urine as early detectors of the disease (uNGAL, uKIM-1) and in relation to albuminuria.	- Endemic participants reported no difference in the uNGAL levels. - Children from endemic regions had higher uKIM-1 expression compared to those from other regions - Albuminuric participants reported elevated uKIM-1 levels compared to normoalbuminuric

## Data Availability

Data sharing not applicable. No new data were created or analyzed in this study. Data sharing is not applicable to this article. Search protocol of studied records is available in Section 2.1 below the Figure 1.

## Abbreviations

AKI	Acute Kidney Injury
ALL	Acute lymphoblastic leukaemia
BMP-7	Blood bone morphogenic protein-7
CAKUT	Congenital anomalies of the kidneys and the urinary tract
CKD	Chronic Kidney Disease
CKiD	Chronic Kidney Disease in Children (CKiD) cohort from 54 North American centres
ESKD	End-stage kidney disease
FGF23	Fibroblast growth factor 23
GFR	Glomerular Filtration Rate
HN	Hydronephrosis
HSP	Heat Shock Protein
IL-18	Interleukin—18
IL-6	Interleukin—6
KDIGO	Kidney Disease: Improving Global Outcomes
KIM-1	Kidney Injury Molecule—1
L-FABP	Liver-type Fatty Acid-Binding Protein
MCP-1	Monocyte Chemotactic Protein—1
MMP	Matrix Metaloproteinase
NAG	N-Acetyl-β-d-amino Glycosidase
NGAL	Neutrophil Gelatinase-Associated Lipocalin
PIIINP	procollagen III N-terminal peptide
RBP4	Retinol-binding Protein 4
SLE	Systemic lupus erythematosus
suPAR	Blood soluble urokinase-type plasminogen activator receptor
TFF3	Treofil Factor 3TGF-β1—Blood transforming growth factor β1
TIMP	Tissue Inhibitor of Metaloproteinase
TNFR 1, 2	Tumor Necrosis Receptor 1 and 2
VDBP	Vitamin D-binding Protein
YKL-40	Chitinase-3-like protein 1

## References

[1] Greenbaum L.A., Warady B.A., Furth S.L. (2009). Current advances in chronic kidney disease in children: Growth, cardiovascular, and neurocognitive risk factors. Semin. Nephrol..

[2] Harshman L.A., Johnson R.J., Matheson M.B., Kogon A.J., Shinnar S., Gerson A.C., Warady B.A., Furth S.L., Hooper S.R. (2019). Academic achievement in children with chronic kidney disease: A report from the CKiD cohort. Pediatr. Nephrol..

[3] Harambat J., van Stralen K.J., Kim J.J., Tizard E.J. (2012). Epidemiology of chronic kidney disease in children. Pediatr. Nephrol..

[4] van Amstel S.P., Noordzij M., Warady B.A., Cano F., Craig J.C., Groothoff J.W., Ishikura K., Neu A., Safouh H., Xu H. (2018). Renal replacement therapy for children throughout the world: The need for a global registry. Pediatr. Nephrol..

[5] Harambat J., Madden I. (2022). What is the true burden of chronic kidney disease in children worldwide?. Pediatr. Nephrol..

[6] Tasic V., Janchevska A., Emini N., Sahpazova E., Gucev Z., Polenakovic M. (2016). Chronic kidney disease—Pediatric risk factors. Prilozi.

[7] Ahn S.Y., Moxey-Mims M. (2018). CKD in Children: The Importance of a National Epidemiologic Study. Am. J. Kidney Dis..

[8] Helal I., Fick-Brosnahan G.M., Reed-Gitomer B., Schrier R.W. (2012). Glomerular hyperfiltration: Definitions, mechanisms and clinical implications. Nat. Rev. Nephrol..

[9] Hogg R.J., Furth S., Lemley K.V., Portman R., Schwartz G.J., Coresh J., Balk E., Lau J., Levin A., Kausz A.T. (2003). National Kidney Foundation’s Kidney Disease Outcomes Quality Initiative clinical practice guidelines for chronic kidney disease in children and adolescents: Evaluation, classification, and stratification. Pediatrics.

[10] Levin A., Stevens P.E. (2014). Summary of KDIGO 2012 CKD Guideline: Behind the scenes, need for guidance, and a framework for moving forward. Kidney Int..

[11] Levey A.S., Cattran D., Friedman A., Miller W.G., Sedor J., Tuttle K., Kasiske B., Hostetter T. (2009). Proteinuria as a surrogate outcome in CKD: Report of a scientific workshop sponsored by the National Kidney Foundation and the US Food and Drug Administration. Am. J. Kidney Dis..

[12] Perrone R.D., Madias N.E., Levey A.S. (1992). Serum creatinine as an index of renal function: New insights into old concepts. Clin. Chem..

[13] Greenberg J.H., Parikh C.R. (2017). Biomarkers for Diagnosis and Prognosis of AKI in Children: One Size Does Not Fit All. Clin. J. Am. Soc. Nephrol..

[14] Parikh C.R., Lu J.C., Coca S.G., Devarajan P. (2010). Tubular proteinuria in acute kidney injury: A critical evaluation of current status and future promise. Ann. Clin. Biochem..

[15] Greenberg J.H., Kakajiwala A., Parikh C.R., Furth S. (2018). Emerging biomarkers of chronic kidney disease in children. Pediatr. Nephrol..

[16] Fathallah-Shaykh S.A. (2017). Proteinuria and progression of pediatric chronic kidney disease: Lessons from recent clinical studies. Pediatr. Nephrol..

[17] Wong C.S., Pierce C.B., Cole S.R., Warady B.A., Mak R.H., Benador N.M., Kaskel F., Furth S.L., Schwartz G.J., CKiD Investigators (2009). Association of proteinuria with race, cause of chronic kidney disease, and glomerular filtration rate in the chronic kidney disease in children study. Clin. J. Am. Soc. Nephrol..

[18] Warady B.A., Abraham A.G., Schwartz G.J., Wong C.S., Muñoz A., Betoko A., Mitsnefes M., Kaskel F., Greenbaum L.A., Mak R.H. (2015). Predictors of Rapid Progression of Glomerular and Nonglomerular Kidney Disease in Children and Adolescents: The Chronic Kidney Disease in Children (CKiD) Cohort. Am. J. Kidney Dis..

[19] Ruiz-Ortega M., Rayego-Mateos S., Lamas S., Ortiz A., Rodrigues-Diez R.R. (2020). Targeting the progression of chronic kidney disease. Nat. Rev. Nephrol..

[20] Zhong J., Yang H.C., Fogo A.B. (2017). A perspective on chronic kidney disease progression. Am. J. Physiol. Renal. Physiol..

[21] Tesch G.H. (2010). Review: Serum and urine biomarkers of kidney disease: A pathophysiological perspective. Nephrology.

[22] Devarajan P. (2010). The use of targeted biomarkers for chronic kidney disease. Adv. Chronic Kidney Dis..

[23] Cagney D.N., Sul J., Huang R.Y., Ligon K.L., Wen P.Y., Alexander B.M. (2018). The FDA NIH Biomarkers, EndpointS, and other Tools (BEST) resource in neuro-oncology. Neuro-Oncology.

[24] Page M.J., McKenzie J.E., Bossuyt P.M., Boutron I., Hoffmann T.C., Mulrow C.D., Shamseer L., Tetzlaff J.M., Akl E.A., Brennan S. (2021). The PRISMA 2020 statement: An updated guideline for reporting systematic reviews. BMJ.

[25] Musiał K., Zwolińska D. (2018). Fractional excretion as a new marker of tubular damage in children with chronic kidney disease. Clin. Chim. Acta Int. J. Clin. Chem..

[26] Greenberg J.H., Devarajan P., Thiessen-Philbrook H.R., Krawczeski C., Parikh C.R., Zappitelli M., TRIBE-AKI Consortium (2018). Kidney injury biomarkers 5 years after AKI due to pediatric cardiac surgery. Pediatr. Nephrol..

[27] Bieniaś B., Sikora P. (2018). Potential Novel Biomarkers of Obstructive Nephropathy in Children with Hydronephrosis. Dis. Markers.

[28] Wu C.Y., Yang H.Y., Chien H.P., Tseng M.H., Huang J.L. (2018). Urinary clusterin-a novel urinary biomarker associated with pediatric lupus renal histopathologic features and renal survival. Pediatr. Nephrol..

[29] Lipiec K., Adamczyk P., Świętochowska E., Ziora K., Szczepańska M. (2018). L-FABP and IL-6 as markers of chronic kidney damage in children after hemolytic uremic syndrome. Adv. Clin. Exp. Med..

[30] Bartoli F., Pastore V., Calè I., Aceto G., Campanella V., Lasalandra C., Magaldi S., Niglio F., Basile A., Cocomazzi R. (2019). Prospective Study on Several Urinary Biomarkers as Indicators of Renal Damage in Children with CAKUT. Eur. J. Pediatr. Surg..

[31] Azukaitis K., Ju W., Kirchner M., Nair V., Smith M., Fang Z., Thurn-Valsassina D., Bayazit A., Niemirska A., Canpolat N. (2019). Low levels of urinary epidermal growth factor predict chronic kidney disease progression in children. Kidney Int..

[32] Musiał K., Zwolińska D. (2019). New markers of cell migration and inflammation in children with chronic kidney disease. Biomarkers.

[33] Taranta-Janusz K., Moczulska A., Nosek H., Michaluk-Skutnik J., Klukowski M., Wasilewska A. (2019). Urinary procollagen III aminoterminal propeptide and β-catenin—New diagnostic biomarkers in solitary functioning kidney?. Adv. Med. Sci..

[34] Nickavar A., Valavi E., Safaeian B., Moosavian M. (2020). Validity of urine neutrophile gelatinase-associated lipocalin in children with primary vesicoureteral reflux. Int. Urol. Nephrol..

[35] Staub E., Urfer-Maurer N., Lemola S., Risch L., Evers K.S., Welzel T., Pfister M. (2020). Comparison of Blood Pressure and Kidney Markers between Adolescent Former Preterm Infants and Term Controls. Children.

[36] Latoch E., Konończuk K., Taranta-Janusz K., Muszyńska-Rosłan K., Szymczak E., Wasilewska A., Krawczuk-Rybak M. (2020). Urine NGAL and KIM-1: Tubular injury markers in acute lymphoblastic leukemia survivors. Cancer Chemother. Pharmacol..

[37] Gul A., Yilmaz R., Ozmen Z.C., Gumuser R., Demir O., Unsal V. (2020). Assessment of renal function in obese and overweight children with NGAL and KIM-1 biomarkers. Valoración de la función renal en niños con sobrepeso y obesidad con las moléculas NGAL y KIM-1. Nutr. Hosp..

[38] McLeod D.J., Sebastião Y.V., Ching C.B., Greenberg J.H., Furth S.L., Becknell B. (2020). Longitudinal kidney injury biomarker trajectories in children with obstructive uropathy. Pediatr. Nephrol..

[39] Greenberg J.H., Abraham A.G., Xu Y., Schelling J.R., Feldman H.I., Sabbisetti V.S., Gonzalez M.C., Coca S., Schrauben S.J., Waikar S.S. (2020). Plasma Biomarkers of Tubular Injury and Inflammation Are Associated with CKD Progression in Children. J. Am. Soc. Nephrol..

[40] Weidemann D.K., Abraham A.G., Roem J.L., Furth S.L., Warady B.A. (2020). Plasma Soluble Urokinase Plasminogen Activator Receptor (suPAR) and CKD Progression in Children. Am. J. Kidney Dis..

[41] Musiał K., Zwolińska D. (2020). Monocyte chemoattractant protein-1, macrophage colony stimulating factor, survivin, and tissue inhibitor of matrix metalloproteinases-2 in analysis of damage and repair related to pediatric chronic kidney injury. Adv. Clin. Exp. Med..

[42] Stabouli S., Kotsis V., Maliachova O., Printza N., Chainoglou A., Christoforidis A., Taparkou A., Dotis J., Farmaki E., Zafeiriou D. (2020). Matrix metalloproteinase-2, -9 and arterial stiffness in children and adolescents: The role of chronic kidney disease, diabetes, and hypertension. Int. J. Cardiol. Hypertens..

[43] Anand S., Bajpai M., Khanna T., Kumar A. (2021). Urinary biomarkers as point-of-care tests for predicting progressive deterioration of kidney function in congenital anomalies of kidney and urinary tract: Trefoil family factors (TFFs) as the emerging biomarkers. Pediatr. Nephrol..

[44] Leibler J.H., Ramirez-Rubio O., Velázquez J.J.A. (2021). Biomarkers of kidney injury among children in a high-risk region for chronic kidney disease of uncertain etiology. Pediatr. Nephrol..

[45] Będzichowska A., Jobs K., Kloc M., Bujnowska A., Kalicki B. (2021). The Assessment of the Usefulness of Selected Markers in the Diagnosis of Chronic Kidney Disease in Children. Biomark. Insights.

[46] Jacobson M.H., Wu Y., Liu M. (2021). Organophosphate pesticides and progression of chronic kidney disease among children: A prospective cohort study. Environ. Int..

[47] Sethi S.K., Sharma R., Gupta A., Tibrewal A., Akole R., Dhir R., Soni K., Bansal S.B., Jha P.K., Bhan A. (2021). Long-Term Renal Outcomes in Children with Acute Kidney Injury Post Cardiac Surgery. Kidney Int. Rep..

[48] Ahn M.B., Cho K.S., Kim S.K., Kim S.H., Cho W.K., Jung M.H., Suh J.S., Suh B.K. (2021). Poor Glycemic Control Can Increase the Plasma Kidney Injury Molecule-1 Concentration in Normoalbuminuric Children and Adolescents with Diabetes Mellitus. Children.

[49] Williams C.E.C., Toner A., Wright R.D., Oni L. (2021). A systematic review of urine biomarkers in children with IgA vasculitis nephritis. Pediatr. Nephrol..

[50] Nosek H., Jankowska D., Brzozowska K., Kazberuk K., Wasilewska A., Taranta-Janusz K. (2021). Tumor Necrosis Factor-Like Weak Inducer of Apoptosis and Selected Cytokines-Potential Biomarkers in Children with Solitary Functioning Kidney. J. Clin. Med..

[51] Sandokji I., Greenberg J.H. (2021). Plasma and Urine Biomarkers of CKD: A Review of Findings in the CKiD Study. Semin. Nephrol..

[52] Turczyn A., Pańczyk-Tomaszewska M., Krzemień G., Górska E., Demkow U. (2021). The Usefulness of Urinary Periostin, Cytokeratin-18, and Endoglin for Diagnosing Renal Fibrosis in Children with Congenital Obstructive Nephropathy. J. Clin. Med..

[53] Tunçay S.C., Doğan E., Hakverdi G., Tutar Z.Ü., Mir S. (2021). Interleukin-8 is increased in chronic kidney disease in children, but not related to cardiovascular disease. J. Bras. Nefrol..

[54] Denburg M.R., Xu Y., Abraham A.G., Coresh J., Chen J., Grams M.E., Feldman H.I., Kimmel P.L., Rebholz C.M., Rhee E.P. (2021). Metabolite Biomarkers of CKD Progression in Children. Clin. J. Am. Soc. Nephrol..

[55] Johnson M.J., Tommerdahl K.L., Vinovskis C., Waikar S., Reinicke T., Parikh C.R., Obeid W., Nelson R.G., van Raalte D.H., Pyle L. (2022). Relationship between biomarkers of tubular injury and intrarenal hemodynamic dysfunction in youth with type 1 diabetes. Pediatr. Nephrol..

[56] Gunasekara T.D.K.S.C., De Silva P.M.C.S., Ekanayake E.M.D.V., Thakshila W.A.K.G., Pinipa R.A.I., Sandamini P.M.M.A., Gunarathna S.D., Chandana E.P.S., Jayasinghe S.S., Herath C. (2022). Urinary biomarkers indicate pediatric renal injury among rural farming communities in Sri Lanka. Sci. Rep..

[57] Alderson H.V., Ritchie J.P., Pagano S., Middleton R.J., Pruijm M., Vuilleumier N., Kalra P.A. (2016). The Associations of Blood Kidney Injury Molecule-1 and Neutrophil Gelatinase-Associated Lipocalin with Progression from CKD to ESRD. Clin. J. Am. Soc. Nephrol..

[58] Gowda S., Desai P.B., Kulkarni S.S., Hull V.V., Math A.A., Vernekar S.N. (2010). Markers of renal function tests. N. Am. J. Med. Sci..

[59] Ferguson M.A., Waikar S.S. (2012). Established and emerging markers of kidney function. Clin. Chem..

[60] Mårtensson J., Xu S., Bell M., Martling C.R., Venge P. (2012). Immunoassays distinguishing between HNL/NGAL released in urine from kidney epithelial cells and neutrophils. Clin. Chim. Acta.

[61] Brunner H.I., Mueller M., Rutherford C., Passo M.H., Witte D., Grom A., Mishra J., Devarajan P. (2006). Urinary neutrophil gelatinase-associated lipocalin as a biomarker of nephritis in childhood-onset systemic lupus erythematosus. Arthritis Rheum..

[62] Trachtman H., Christen E., Cnaan A., Patrick J., Mai V., Mishra J., Jain A., Bullington N., Devarajan P. (2006). Investigators of the HUS-SYNSORB Pk Multicenter Clinical Trial. Urinary neutrophil gelatinase-associated lipocalcin in D+HUS: A novel marker of renal injury. Pediatr. Nephrol..

[63] Smith E.R., Lee D., Cai M.M., Tomlinson L.A., Ford M.L., McMahon L.P., Holt S.G. (2013). Urinary neutrophil gelatinase-associated lipocalin may aid prediction of renal decline in patients with non-proteinuric Stages 3 and 4 chronic kidney disease (CKD). Nephrol. Dial. Transplant..

[64] Liu K.D., Yang W., Go A.S., Anderson A.H., Feldman H.I., Fischer M.J., He J., Kallem R.R., Kusek J.W., Master S.R. (2015). Urine neutrophil gelatinase-associated lipocalin and risk of cardiovascular disease and death in CKD: Results from the Chronic Renal Insufficiency Cohort (CRIC) Study. Am. J. Kidney Dis..

[65] Kasahara M., Mori K., Satoh N., Kuwabara T., Yokoi H., Shimatsu A., Sugawara A., Mukoyama M., Nakao K. (2009). Reduction in urinary excretion of neutrophil gelatinase-associated lipocalin by angiotensin receptor blockers in hypertensive patients. Nephrol. Dial. Transplant..

[66] Kuwabara T., Mori K., Mukoyama M., Kasahara M., Yokoi H., Saito Y., Yoshioka T., Ogawa Y., Imamaki H., Kusakabe T. (2009). Urinary neutrophil gelatinase-associated lipocalin levels reflect damage to glomeruli, proximal tubules, and distal nephrons. Kidney Int..

[67] Gracie J.A., Robertson S.E., McInnes I.B. (2003). Interleukin-18. J. Leukoc. Biol..

[68] Liu X., Guan Y., Xu S., Li Q., Sun Y., Han R., Jiang C. (2016). Early Predictors of Acute Kidney Injury: A Narrative Review. Kidney Blood Press Res..

[69] Zubowska M., Wyka K., Fendler W., Młynarski W., Zalewska-Szewczyk B. (2013). Interleukin 18 as a marker of chronic nephropathy in children after anticancer treatment. Dis. Markers.

[70] Hall I.E., Doshi M.D., Reese P.P., Marcus R.J., Thiessen-Philbrook H., Parikh C.R. (2012). Association between peritransplant kidney injury biomarkers and 1-year allograft outcomes. Clin. J. Am. Soc. Nephrol..

[71] Shlipak M.G., Scherzer R., Abraham A., Tien P.C., Grunfeld C., Peralta C.A., Devarajan P., Bennett M., Butch A.W., Anastos K. (2012). Urinary markers of kidney injury and kidney function decline in HIV-infected women. J. Acquir. Immune. Defic. Syndr..

[72] Peralta C., Scherzer R., Grunfeld C., Abraham A., Tien P., Devarajan P., Bennett M., Butch A., Anastos K., Cohen M. (2014). Urinary biomarkers of kidney injury are associated with all-cause mortality in the Women’s Interagency HIV Study (WIHS). HIV Med..

[73] Yin C., Wang N. (2016). Kidney injury molecule-1 in kidney disease. Ren. Fail..

[74] Sandokji I., Greenberg J.H. (2020). Novel biomarkers of acute kidney injury in children: An update on recent findings. Curr. Opin. Pediatr..

[75] Han W.K., Bailly V., Abichandani R., Thadhani R., Bonventre J.V. (2002). Kidney Injury Molecule-1 (KIM-1): A novel biomarker for human renal proximal tubule injury. Kidney Int..

[76] Bieniaś B., Zajączkowska M., Borzęcka H., Sikora P., Wieczorkiewicz-Płaza A., Wilczyńska B. (2015). Early Markers of Tubulointerstitial Fibrosis in Children With Idiopathic Nephrotic Syndrome: Preliminary Report. Medicine.

[77] Ucakturk A., Avci B., Genc G., Ozkaya O., Aydin M. (2016). Kidney injury molecule-1 and neutrophil gelatinase associated lipocalin in normoalbuminuric diabetic children. J. Pediatr. Endocrinol. Metab..

[78] Tang J., Liu N., Zhuang S. (2013). Role of epidermal growth factor receptor in acute and chronic kidney injury. Kidney Int..

[79] Norman J., Tsau Y.K., Bacay A., Fine L.G. (1990). Epidermal growth factor accelerates functional recovery from ischaemic acute tubular necrosis in the rat: Role of the epidermal growth factor receptor. Clin. Sci..

[80] Ju W., Nair V., Smith S., Zhu L., Shedden K., Song P.X.K., Mariani L.H., Eichinger F.H., Berthier C.C., Randolph A. (2015). Tissue transcriptome-driven identification of epidermal growth factor as a chronic kidney disease biomarker. Sci. Transl. Med..

[81] Penders J., Delanghe J.R. (2004). Alpha 1-microglobulin: Clinical laboratory aspects and applications. Clin. Chim, Act..

[82] O’Seaghdha C.M., Hwang S.J., Larson M.G., Meigs J.B., Vasan R.S., Fox C.S. (2013). Analysis of a urinary biomarker panel for incident kidney disease and clinical outcomes. J. Am. Soc. Nephrol..

[83] Jotwani V., Scherzer R., Abraham A. (2015). Association of urine α1-microglobulin with kidney function decline and mortality in HIV-infected women. Clin. J. Am. Soc. Nephrol..

[84] Al-Lamki R.S., Mayadas T.N. (2015). TNF receptors: Signaling pathways and contribution to renal dysfunction. Kidney Int..

[85] Mehaffey E., Majid D.S.A. (2017). Tumor necrosis factor-α, kidney function, and hypertension. Am. J. Physiol. Renal Physiol..

[86] Guo G., Morrissey J., McCracken R., Tolley T., Klahr S. (1999). Role of TNFR1 and TNFR2 receptors in tubulointerstitial fibrosis of obstructive nephropathy. Am. J. Physiol..

[87] Ernandez T., Mayadas T.N. (2009). Immunoregulatory role of TNF alpha in inflammatory kidney diseases. Kidney Int..

[88] Shankar A., Sun L., Klein B.E., Lee K.E., Muntner P., Nieto F.J., Tsai M.Y., Cruickshanks K.J., Schubert C.R., Brazy P.C. (2011). Markers of inflammation predict the long-term risk of developing chronic kidney disease: A population-based cohort study. Kidney Int..

[89] Jutley R.S., Youngson G.G., Eremin O., Ninan G.K. (2000). Serum cytokine profile in reflux nephropathy. Pediatr. Surg. Int..

[90] Moreira J.M., da Silva A.N., Marciano Vieira É.L., Teixeira A.L., Kummer A.M., Simões E., Silva A.C. (2019). Soluble tumor necrosis factor receptors are associated with severity of kidney dysfunction in pediatric chronic kidney disease. Pediatr. Nephrol..

[91] Hayek S.S., Sever S., Ko Y.A., Trachtman H., Awad M., Wadhwani S., Altintas M.M., Wei C., Hotton A.L., French A.L. (2015). Soluble Urokinase Receptor and Chronic Kidney Disease. N. Engl. J. Med..

[92] Wei C., El Hindi S., Li J., Fornoni A., Goes N., Sageshima J., Maiguel D., Karumanchi S.A., Yap H.K., Saleem M. (2011). Circulating urokinase receptor as a cause of focal segmental glomerulosclerosis. Nat. Med..

[93] Wei C., Trachtman H., Li J., Dong C., Friedman A.L., Gassman J.J., McMahan J.L., Radeva M., Heil K.M., Trautmann A. (2012). Circulating suPAR in two cohorts of primary FSGS. J. Am. Soc. Nephrol..

[94] Schaefer F., Trachtman H., Wühl E., Kirchner M., Hayek S.S., Anarat A., Duzova A., Mir S., Paripovic D., Yilmaz A. (2017). Association of Serum Soluble Urokinase Receptor Levels With Progression of Kidney Disease in Children. JAMA Pediatr..

[95] Kim M.J., Tam F.W. (2011). Urinary monocyte chemoattractant protein-1 in renal disease. Clin. Chim. Acta.

[96] Ghobrial E.E., El Hamshary A.A., Mohamed A.G., Abd El Raheim Y.A., Talaat A.A. (2015). Urinary monocyte chemoattractant protein-1 as a biomarker of lupus nephritis activity in children. Saudi J. Kidney Dis. Transpl..

[97] Vianna H.R., Soares C.M., Silveira K.D., Elmiro G.S., Mendes P.M., de Sousa Tavares M., Teixeira M.M., Miranda D.M., Simões E Silva A.C. (2013). Cytokines in chronic kidney disease: Potential link of MCP-1 and dyslipidemia in glomerular diseases. Pediatr. Nephrol..

[98] Schmidt I.M., Hall I.E., Kale S. (2013). Chitinase-like protein Brp-39/YKL-40 modulates the renal response to ischemic injury and predicts delayed allograft function. J. Am. Soc. Nephrol..

[99] Tsakas S., Goumenos D.S. (2006). Accurate measurement and clinical significance of urinary transforming growth factor-beta1. Am. J. Nephrol..

[100] Cheng O., Thuillier R., Sampson E. (2006). Connective tissue growth factor is a biomarker and mediator of kidney allograft fibrosis. Am. J. Transplant..

[101] Woroniecki R.P., Shatat I.F., Supe K., Du Z., Kaskel F.J. (2008). Urinary cytokines and steroid responsiveness in idiopathic nephrotic syndrome of childhood. Am. J. Nephrol..

[102] Zieg J., Blahova K., Seeman T., Bronsky J., Dvorakova H., Pechova M., Janda J., Matousovic K. (2011). Urinary transforming growth factor-β1 in children with obstructive uropathy. Nephrology.

[103] Musiał K., Fornalczyk K., Zwolińska D. (2008). Osteopontin (OPN), PDGF-BB (platelet-derived growth factor) and BMP-7 (bone morphogenetic protein) as markers of atherogenesis in children with chronic kidney disease (CKD) treated conservatively--preliminary results. Pol. Merkur. Lek..

[104] Hruska K.A., Guo G., Wozniak M. (2000). Osteogenic protein-1 prevents renal fibrogenesis associated with ureteral obstruction. Am. J. Physiol.-Renal Physiol..

[105] Zeisberg M., Hanai J., Sugimoto H., Mammoto T., Charytan D., Strutz F., Kalluri R. (2003). BMP-7 counteracts TGF-beta1-induced epithelial-to-mesenchymal transition and reverses chronic renal injury. Nat. Med..

[106] Wiercinska E., Naber H.P.H., Pardali E., Pluijm G.V.D., Dam H.V., Dijke P.T. (2011). The TGF-β/Smad pathway induces breast cancer cell invasion through the up-regulation of matrix metalloproteinase 2 and 9 in a spheroid invasion model system. Breast Cancer Res. Treat..

[107] Musiał K., Zwolińska D. (2011). Matrix metalloproteinases (MMP-2,9) and their tissue inhibitors (TIMP-1,2) as novel markers of stress response and atherogenesis in children with chronic kidney disease (CKD) on conservative treatment. Cell Stress Chaperones.

[108] Korzeniecka-Kozerska A., Wasilewska A., Tenderenda E., Sulik A., Cybulski K. (2013). Urinary MMP-9/NGAL ratio as a potential marker of FSGS in nephrotic children. Dis. Markers.

[109] Ghoul B.E., Squalli T., Servais A., Elie C., Meas-Yedid V., Trivint C., Vanmassenhove J., Grünfeld J.P., Olivo-Marin J.C., Thervet E. (2010). Urinary procollagen III aminoterminal propeptide (PIIINP): A fibrotest for the nephrologist. Clin. J. Am. Soc. Nephrol..

[110] Teppo A.M., Törnroth T., Honkanen E., Grönhagen-Riska C. (2003). Urinary amino-terminal propeptide of type III procollagen (PIIINP) as a marker of interstitial fibrosis in renal transplant recipients. Transplantation.

[111] Portale A.A., Wolf M., Jüppner H., Messinger S., Kumar J., Wesseling-Perry K., Schwartz G.J., Furth S.L., Warady B.A., Salusky I.B. (2014). Disordered FGF23 and mineral metabolism in children with CKD. Clin. J. Am. Soc. Nephrol..

[112] Portale A.A., Wolf M.S., Messinger S., Perwad F., Jüppner H., Warady B.A., Furth S.L., Salusky I.B. (2016). Fibroblast Growth Factor 23 and Risk of CKD Progression in Children. Clin. J. Am. Soc. Nephrol..

[113] Kamijo-Ikemori A., Sugaya T., Obama A., Hiroi J., Miura H., Watanabe M., Kumai T., Ohtani-Kaneko R., Hirata K., Kimura K. (2006). Liver-type fatty acid-binding protein attenuates renal injury induced by unilateral ureteral obstruction. Am. J. Pathol..

[114] Kamijo A., Sugaya T., Hikawa A., Yamanouchi M., Hirata Y., Ishimitsu T., Numabe A., Takagi M., Hayakawa H., Tabei F. (2006). Urinary liver-type fatty acid binding protein as a useful biomarker in chronic kidney disease. Mol. Cell Biochem..

[115] Jaconi S., Rose K., Hughes G.J., Saurat J.H., Siegenthaler G. (1995). Characterization of two post-translationally processed forms of human serum retinol-binding protein: Altered ratios in chronic renal failure. J. Lipid Res..

[116] Fathallah-Shaykh S.A., Flynn J.T., Pierce C.B., Abraham A.G., Blydt-Hansen T.D., Massengill S.F., Moxey-Mims M.M., Warady B.A., Furth S.L., Wong C.S. (2015). Progression of Pediatric CKD of Nonglomerular Origin in the CKiD Cohort. Clin. J. Am. Soc. Nephrol..

